# Retinal Pigment Epithelium Transplantation in Retinal Disease: Clinical Trial Development, Challenges, and Future Directions

**DOI:** 10.3390/biom15081167

**Published:** 2025-08-15

**Authors:** Qin Chen, Ting Zhang, Zhi Chen, Jingwen Zeng, Aine O’Connor, Meidong Zhu, Mark C. Gillies, Fang Lu, Ling Zhu

**Affiliations:** 1 The Department of Ophthalmology, West China Hospital, Sichuan University, Chengdu 610041, China; 2 Save Sight Institute, University of Sydney, Sydney, NSW 2015, Australia; 3 Intelligent Polymer Research Institute, University of Wollongong, Wollongong, NSW 2522, Australia; 4 New South Wales Tissue Bank, Sydney Eye Hospital, New South Wales Organ and Tissue Donation Service, Sydney, NSW 2000, Australia

**Keywords:** retinal pigment epithelium (RPE) transplantation, retinal degenerative diseases, stem cell-derived RPE, clinical trials, cell therapy

## Abstract

Replacement of the retinal pigment epithelium (RPE) is emerging as a promising approach to treat degenerative retinal diseases, including age-related macular degeneration and Stargardt disease, in which RPE function cannot otherwise be restored. Despite the limitations of existing treatments, advances in cell sourcing and surgical methods have enabled initial human trials of RPE transplantation, with early results indicating potential efficacy. This review comprehensively examines the evolution of RPE transplantation in recent decades, highlighting the advantages and limitations of different cell sources and delivery methods. Current clinical trial data are analyzed with a particular focus on immune rejection risks, surgical complications, and long-term safety. Despite encouraging safety profiles, achieving consistent and sustained visual improvement remains a challenge, as vision outcomes might be influenced by factors such as disease stage at intervention, transplantation site, number of cells transplanted, and duration of follow-up. Key challenges, such as cell or graft survival and integration with the host retina, are discussed in depth, as overcoming these obstacles is essential for achieving stable and effective RPE replacement. Future research directions, including innovations in biomaterials, molecular modification strategies, and personalized approaches, hold promise for enhancing the efficacy and durability of RPE transplantation for retinal disease.

## 1. Introduction

The retinal pigment epithelium (RPE) is a monolayer of pigmented cells situated between the photoreceptors and the choroid that plays a crucial role in retinal health. Functions of the RPE include (1) maintaining the visual cycle by transporting and storing retinoids, (2) phagocytosing and degrading photoreceptor outer segments, (3) absorbing light and safeguarding the retina from photooxidative damage, (4) facilitating the transport of nutrients, ions, and water while acting as a blood–retina barrier, and (5) secreting essential factors to maintain immune privilege [1]. The underlying pathological mechanisms of RPE dysfunction often involve oxidative stress, chronic inflammation, and disruptions in autophagy, all of which lead to cellular dysfunction or death [2]. Preserving or restoring RPE function is critical for sustaining retinal integrity and preventing further loss of vision.

RPE dysfunction is a critical factor in the pathogenesis of several retinal diseases. In age-related macular degeneration (AMD), RPE degeneration leads to photoreceptor loss, manifesting as progressive impairment of central vision [3]. Stargardt disease (STGD) is caused by mutations in the *ABCA4* gene, which result in the accumulation of toxic lipofuscin within the RPE that causes early-onset vision loss [4]. RPE dysfunction is also a feature of many other retinal conditions, such as retinitis pigmentosa, and certain choroidal dystrophies [5,6].

Current treatments, which often aim to mitigate damage or slow disease progression, have significant limitations. For example, anti-VEGF therapy offers limited improvement in long-term vision, while anti-complement therapy has minimal visual benefit. Both require frequent intravitreal injections [7,8]. Gene therapy is limited by high cost and inefficacy in advanced disease. The ability of these approaches to restore the lost RPE functionality is limited. RPE transplantation has emerged as a promising strategy to address this gap to potentially halt disease progression by replacing damaged cells and restoring critical functions.

**Figure 1:**
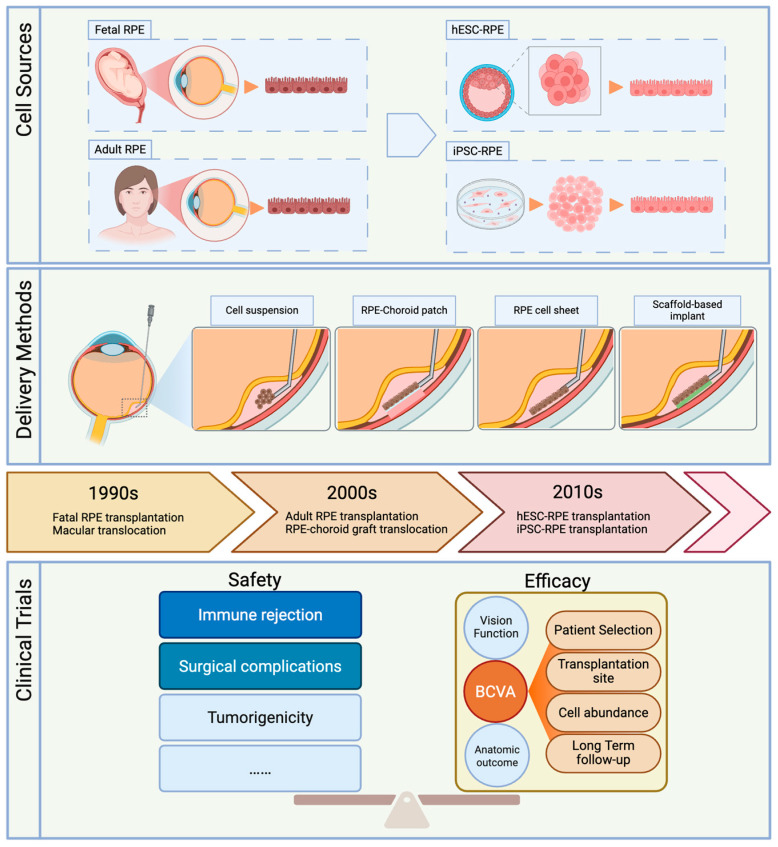
A schematic diagram illustrating the development of RPE transplantation, highlighting advancements in cell sources, surgical delivery techniques, and characteristics of associated clinical trials.

This review examines the evolution of RPE transplantation, compares various delivery techniques and analyzes recent clinical trials. We highlight both the successes and limitations of these approaches, with a focus on clinical outcomes and adverse effects over time. Critical challenges such as cell or graft survival, integration with the host retina, and immune rejection are discussed in detail, as addressing these issues is essential for achieving stable and effective RPE replacement therapies (Figure 1).

## 2. History and Development of RPE Transplantation

### 2.1. Cell Source Development

Researchers have explored the feasibility of RPE transplantation in patients with AMD since the 1990s. Initial therapeutic outcomes were, however, suboptimal. Peyman et al. were the first to report autologous and homologous RPE transplantation in two patients with end-stage AMD. At the same time, other clinicians conducted transplants using allogeneic fetal RPE cells in five patients [9,10]. These early clinical attempts marked a significant transition, shifting RPE transplantation from preclinical animal models to clinical applications.

Around the same period, clinicians attempted macular translocation surgery as an indirect approach to RPE transplantation. This procedure is typically used to treat conditions like wet AMD or myopic choroidal neovascularization. The objective of this procedure was to preserve or enhance central vision by repositioning the macula to an area with healthier RPE and choroid [11,12,13]. However, due to considerable complications, including retinal detachment, proliferative vitreoretinopathy, and an unfavorable risk–benefit ratio, macular translocation was eventually discontinued after its initial clinical evaluation [14].

RPE transplantation has evolved since the early 2000s to include transplantation of autologous RPE cells and RPE–choroid tissues. Binder et al. pioneered a technique in which RPE cells were harvested from the nasal subretinal area of the same AMD-affected eye and subsequently transplanted following subretinal membrane excision [15,16]. Although the grafts remained stable for at least 36 months, approximately 50% of patients showed improvement in visual acuity (VA) after a follow-up of 12 to 17 months [17]. Recognizing the critical role of the RPE–choroid complex in maintaining visual function, van Meurs and Joussen et al. introduced the transplantation of autologous peripheral full-thickness grafts of RPE and choroid, strategically positioned beneath the macula in AMD patients with varying types of disease [18,19]. Visual improvement with a gain in vision of at least 15 letters was reported in 78% of treated eyes in a 2 to 10-year follow-up. Significant improvement in vision enhancement was mainly observed in cases with choroidal neovascular membranes and intact external limiting membranes, whereas patients with atrophic maculopathies experienced only temporary visual benefits [20].

Although autologous RPE transplantation has shown promising results, its widespread application remains limited by challenges such as restricted donor sources and the complexity of the surgical procedures. However, with advances in cell engineering technology, RPE transplantation has entered the stem cell era, offering new possibilities for the cell-based treatment of retinal diseases. Functional RPE cells can be derived from multiple lines of human embryonic stem cells (hESCs) and human induced pluripotent stem cells (iPSCs) [21]. Compared to native RPE cells, hESCs derived RPE (hESC-RPE) and iPSCs derived RPE (iPSC-RPE) offer an unlimited supply and potential for genetic modification, making them attractive alternatives for therapeutic applications.

The earliest published hESC-RPE used in clinical trials was MA09, derived from hESC cell lines [22], which was initially tested in pigmented dystrophic RCS rats and ELOVL4 transgenic mice, models for AMD and STGD. The transplanted cells preserved visual function and photoreceptor integrity for over 60 days and were well-tolerated. No gross or microscopic evidence of tumor formation was observed throughout the animals’ lifespans [23]. A landmark clinical trial in humans by Schwartz et al. [22] demonstrated the feasibility of hESC-derived RPE transplantation in patients with AMD and STGD. Over a four-year follow-up period, the study confirmed the safety of hESC-RPE transplantation, with no reported incidences of tumor formation or severe immune rejection [24,25]. Since then, additional hESC-RPE products, such as RG6501, CPCB-RPE, and CTS-hESC-RPE, have entered clinical trials, supported by promising results from preclinical animal studies [26,27]. However, hESC are banned in some countries because they are derived from embryos.

To address the ethical concerns and regulatory barriers associated with the use of ESCs, iPSC-RPE has emerged as a trend source for RPE transplantation. The use of iPSC-RPE also allows for the generation of patient-specific cell lines, enabling autologous transplantation. This approach theoretically reduces the risk of immune rejection and may eliminate the need for long-term immunosuppressive therapy. Takahashi et al. transplanted iPSC-RPE generated from human fibroblasts into the subretinal space of RCS rats. They reported that the grafts promoted restoration of the outer nuclear layer above the implantation site and significantly improved visual function within 9 weeks postoperatively. In a parallel study using the same iPSC-RPE in non-human primates, no signs of immune rejection or tumor formation were observed over a one-year follow-up period [28]. Building on these findings, the same research group conducted a pioneering study transplanting autologous iPSC-derived RPE into an AMD patient without the use of immunosuppressive therapy. Both short-term and long-term observations revealed that the transplanted iPSC-derived RPE sheet survived for four years and appeared to support the surrounding photoreceptors and choroidal vasculature [29]. To date, six clinical trials involving iPSC-RPE transplantation have been registered since 2017, marking a significant advance in the field of regenerative retinal therapies.

Table 1 provides a concise summary of the various cell sources utilized in clinical trials, highlighting their respective advantages and limitations. Currently, research on the genetic modification of iPSC-derived RPE cells is progressing rapidly. Genetically modified RPE cells may exhibit enhanced survival following transplantation and reduced immunogenicity [30]. In parallel, in countries with well-established organ donation systems and supportive populations, native RPE cells can be obtained from donated human eyes through collaboration with local eye banks. These primary RPE cells, after appropriate processing and modification, represent a promising and biologically authentic cell source for transplantation. However, successful implementation depends on overcoming key logistical challenges, including optimizing donor selection and minimizing death-to-retrieval time to ensure cell viability. Eye banks play a vital role in this process by providing access to ethically sourced RPE tissue along with relevant donor information under approved research protocols. Strategic partnerships between researchers and eye banks are essential to unlock the full potential of human donor tissue in advancing RPE transplantation therapies.

### 2.2. Comparison of Delivery Methods

Various surgical approaches have been explored throughout the history of cell transplantation for macular degeneration, including intravitreal injection [31] and suprachoroidal injection [32]. However, the most widely adopted and effective approach remains the subretinal transplantation of cell suspensions or patches, enabling direct interaction between the transplanted cells, photoreceptors, and the underlying choroid. Several techniques are currently used to deliver RPE cells into the subretinal space, including injections of cell suspensions, RPE–choroid patch transplants, RPE cell sheets without material support, and scaffold-based implants. The unique advantages and challenges of each method are summarized in Table 2.

#### 2.2.1. Delivery of Cell Suspensions

In the cell suspension approach, RPE cells are delivered as individual, unorganized cells in a liquid medium via subretinal injection. This method offers a simpler and minimally invasive surgical procedure compared to other delivery techniques [24]. Preclinical studies have demonstrated that suspended cells can form a monolayer in the subretinal space and express functional proteins within four weeks post-injection [33]. The injected RPE cells can disperse widely across the retina, potentially covering a larger area and benefiting patients with diffuse retinal diseases. However, a portion of the cells was also observed to aggregate into clumps and migrate into the inner retina [23,33]. Additionally, cell suspensions carry the risk of transplanted RPE cells localizing within the vitreous or on the surface of the retina. Sung et al. found attached pigmented epithelial cells with a female donor’s chromosomes, XX, in the epiretinal membrane from a male recipient with XY [34]. This may increase the risk of proliferative vitreoretinopathy and potentially reduce the efficacy of treatment [22].

#### 2.2.2. RPE–choroid Patch Transplantation

The RPE–choroid patch technique involves harvesting a small patch of RPE cells along with the underlying Bruch’s membrane and choroid from the peripheral retina, which is then transplanted into the damaged macular region. This approach offers a better cell source, providing an improved chance of restoring normal retinal structures and VA [35,36]. Aged Bruch’s membrane in AMD patients cannot facilitate RPE adhesion well [37]. So, the complex patch structure may help to resolve adhesion challenges and enhance blood flow in the choriocapillaris. However, this surgical method is technically demanding and can cause significant retinal damage. Additionally, postoperative complications, including recurrence of choroidal neovascularization (CNV), are reportedly higher compared to other delivery methods [20].

#### 2.2.3. RPE Cell Sheet Implants

RPE cell sheet implants are generated by culturing stem cell-derived RPE cells in vitro to form a monolayer of polarized and regularly organized cells, which may be more likely to survive in the subretinal space. Due to the absence of physical support, the cell sheet is fragile during implantation, limiting the possibility of forming large grafts. The grafts used in current clinical trials are approximately half the size of membrane-supported RPE grafts, measuring around 1.3 × 3 mm compared to 3 × 6 mm [38]. A notable advantage of this method is the absence of foreign materials, reducing concerns related to safety and immune response [29]. It has been demonstrated that small iPSC-RPE strips can be safely delivered to the target area with minimal surgical invasion, facilitating better expansion and integration into the region of damaged RPE [39,40].

#### 2.2.4. Scaffold-Based RPE Implants

Scaffold-based RPE implantation has emerged as a promising approach, driven by advances in biomaterials. Scaffolds mimic the extracellular matrix, offering enhanced support and an optimized microenvironment for transplanted RPE cells. This method promotes cell survival and integration, ensuring the functional maintenance of the transplanted RPE cells. Furthermore, scaffolds could act as physical barriers to change the choriocapillaris perfusion beneath the implant and may prevent the infiltration of choroidal blood vessels into the retina [41]. Primary RPE, iPSC-RPE, and hESC-RPE are the most widely studied cell types for scaffold-based RPE implants [42,43,44]. Although small and mid-sized animal models such as rats and minipigs are commonly used [44,45], a recent study in cynomolgus monkeys has provided more clinically relevant insights, demonstrating successful anatomical integration and functional restoration in primates [46]. Materials such as polyethylene terephthalate and parylene C have been used in Phase I/II clinical trials, demonstrating limited yet encouraging safety and efficacy outcomes [42,47,48]. Kashani et al. introduced a tightly spaced scaffold design that mimics the architecture of Bruch’s membrane, thereby enhancing nutrient diffusion and cellular adherence [47]. Additionally, scaffold-based implants allow for easy monitoring using optical coherence tomography (OCT). However, these techniques require extensive implant preparation and involve complex surgical procedures. Potential drawbacks include disruptions to oxygen permeability and the induction of inflammatory responses, which must be carefully addressed [49]. Emerging biodegradable biomaterials, such as PLGA (ploy (lactic-co-glycolic acid)) and PCL (Polycaprolactone), have shown promising results in the preclinical research phase. RPE implants constructed using these scaffolds have demonstrated the ability to rescue photoreceptors and improve VA in rat models of retinal degeneration [50]. Several challenges remain in the application of these biomaterials. For instance, certain biomaterials generate acidic degradation byproducts, which can reduce the pH of the surrounding microenvironment and potentially trigger inflammatory responses [51]. Moreover, its degradation kinetics may be difficult to control, potentially resulting in a mismatch with the rate of extracellular matrix regeneration [52]. Natural biopolymer scaffolding, like Collagen-based scaffolds or human amniotic membrane (hAM), has also begun to be used in preclinical trials [53].

Emerging advances have focused on novel delivery methods and minimally invasive techniques to reduce infections and improve the low success rates of cell engraftment. Notably, Wei et al. introduced an injectable microgel system based on gelatin-methacryloyl and hyaluronic acid that notably improved visual outcomes in rat models [54]. Similarly, a mechanically adaptable, biodegradable nanosheet combining polycaprolactone, collagen, and RPE cells has been delivered to rats using a minimally invasive procedure, successfully promoting retinal regeneration [55].

## 3. Overview and Analysis of Clinical Trials

More than 30 clinical trials on RPE transplantation are currently being conducted worldwide. Based on published findings, the safety profile of this treatment with regard to tumorigenesis has been well established. To comprehensively assess the progress and outcomes of RPE transplantation, we systematically collected data from registered clinical trials and published research articles.

A thorough search for clinical trials and published studies on RPE transplantation in retinal diseases was conducted using two major databases. A query on ClinicalTrials.gov, with the keywords “retinal disease, retinal pigment epithelium, transplantation”, yielded 113 results. After excluding irrelevant entries, a total of 31 relevant clinical trials were identified. Similarly, a search on PubMed using the terms “retinal pigment epithelium AND transplantation OR implantation OR translocation,” filtered by article types such as clinical trials, case reports, and clinical investigations, published between 1992 and 2024, following the exclusion of non-relevant publications, yielded 39 articles considered pertinent to this review.

These searches provide a comprehensive overview of the current landscape of research in RPE transplantation, offering valuable insights into ongoing clinical efforts and the existing scientific literature. In this review, we synthesize findings from the clinical trials with detailed published outcomes, summarizing their vision efficacy, safety profiles, and reported side effects (Table 3).

**Table 3:** Clinical trials of RPE transplantation.

Clinical Trial ID	Target Disease	Phase	Actual Enrollment/Study Eyes	Cell Source	Autologous or Allogeneic	Delivery Method	Cell Amount	Delivery Position	Follow-Up Duration	Vision Outcome	Vision Function	Ocular Adverse Effect	References
None	Dry and Wet AMD	NA	84/88	RPE–choroid patch	Autologous	RPE–choroid patch	N/A	Over the fovea	2–10 years	38% gained > 15 letters; 10% lost > 15 letters; rest stable	MP showed central fixation	RD, recurrent CNV, ERM, MH, hemorrhage, macular atrophy	[20]
NCT00401713	Wet AMD	I	7/7	RPE–choroid sheet	Autologous	RPE–choroid patch	>1500	Over the fovea	2 years	5/7 patients with BCVA improved or stable	MP showed more retinal fixation and stable	PVR, RD, ERM	[56]
			7/7	RPE cells	Autologous	Cell suspension	>1500	Over the fovea	2 years	6/7 patients with BCVA improved or stable	MP showed less retinal fixation and stable	ERM	[56]
NCT01344993	Dry AMD	I/II	9/9	hESC-derived RPE (MA09-hRPE)	Allogeneic	Cell suspension	50,000 100,000 150,000 (0.15 mL)	Between atrophic and healthy retina	4 years	BCVA stable or improved	Not mentioned	Cataract, vitreous inflammation	[22,24]
NCT01345006	STGD	I/II	9/9	hESC-derived RPE (MA09-hRPE)	Allogeneic	Cell suspension	50,000 100,000 150,000 (0.15 mL)	Between atrophic and healthy retina	4 years	BCVA stable or improved; one eye worsened	Not mentioned	Cataract, vitreous inflammation, endophthalmitis	[22,24]
NCT01469832	STGD type 1	I/II	12/12	hESC-derived RPE (MA09-hRPE)	Allogeneic	Cell suspension	50,000 100,000 150,000 200,000 (0.15 mL)	Normal areas near vascular arch	1 year	No significant BCVA improvement	No significant change in MP, mfERG, color vision	Subretinal/vitreous hemorrhage, ERM, preretinal/vitreous pigmentation	[57]
NCT01625559	STGD	I	3/3	hESC-derived RPE (MA09-hRPE)	Allogeneic	Cell suspension	50,000 (0.15 mL)	Relatively normal areas near macula	3 years	BCVA improved >3 letters	No significant change in VF, ffERG, mfERG	Retinal hemorrhage, ERM, CNV, RRD, mild immune suppression effects	[58,59]
NCT01674829	Dry AMD	I	2/2	hESC-derived RPE (MA09-hRPE)	Allogeneic	Cell suspension	50,000 (0.15 mL)	Relatively normal areas near macula	1 year	BCVA stable or improved	No significant change in VF, ERG, mfERG	Retinal hemorrhage, ELM, CNV, IOP elevation	[58]
NCT01691261 NCT03102138	Wet AMD	I	2/2	hESC derived RPE (PF-05206388)	Allogeneic	Scaffold-based implant	100,000 (3 × 6 mm)	Over the fovea	5 years	BCVA improved 21–29 letters in 12 months, maintained for 2 years	MP improved short-term but declined; ERG, EOG showed photoreceptor reduction; reading speed improved	PVR-associated traction RD, epiretinal bands, macular traction, retinal thinning	[48,60]
NCT02286089	Dry AMD and GA	I/IIa	24/24	hESC-derived RPE (OpRegen)	Allogeneic	Cell suspension	50,000–200,000	Not mentioned	1–5 years	75% vision improvement or maintenance in Cohort 4	Not mentioned	ERM, RD, CNV (suprachoroidal route)	[61,62]
NCT02590692	Dry AMD and GA	I/IIa	16/16	hESC-derived RPE (CPCB-RPE1)	Allogeneic	Scaffold-based implant	100,000 (3.5 × 6.25 mm)	Over the GA and the fovea	3 years	Treated eyes improved >5 letters and worsened <5 letters in BCVA	MP and mfERG unreliable	Retinal hemorrhage, edema, RD (surgery-related)	[42,47,63]
NCT02749734	STGD type 1	I	7/7	hESC-derived RPE (CTS-hESC-RPE)	Allogeneic	Cell suspension	100,000 (0.1 mL)	Relatively normal areas near macula	5 years	No significant BCVA improvement	No significant change in VF, fVEP, ffERG, mfERG	No severe ocular AE	[64]
	Wet AMD	I	3/3	hESC-derived RPE (CTS-hESC-RPE)	Allogeneic	Cell suspension	1,000,000 (0.1 mL)	Over the fovea	1 year	BCVA improved > 10 letters (untreated eye improved less)	mfERG showed the response density was slightly increased; fVEP kept stable	No ocular severe AE.	[65]
NCT02903576	STGD type 3	I	12/12	hESC-derived RPE (WA-099)	Allogeneic	Cell suspension	1,000,000 (0.1 mL)	Over the fovea	1 year	No significant BCVA improvement	No difference in VF, mfERG, ffERG	Cataract, epiretinal pigmented clumps, retinal atrophy	[66]
NCT03969154	RP	I	7/7	hESC-derived RPE	Allogeneic	Scaffold-based implant	14.5 mm^2^, 4800–15,000 cells/mm^2^	Not mentioned	Not mentioned	Not mentioned	Not mentioned	Not mentioned	[67]
UMIN000011929	Wet AMD	I	2/1	iPSC-derived RPE	Autologous	RPE cell sheet	Around 50,000 (1.3 × 3.0 mm)	Over the fovea	4 years	No significant improvement	MP unchanged	Temporary IOP elevation	[29,38]
UMIN000026003	Wet AMD	I	5/5	iPSC-derived RPE	Allogeneic (HLA matched)	Cell suspension and anti-VEGF	250,000 (0.05 mL)	Relatively normal areas near macula	1 year	No significant improvement	ERG and mfERG mildly decreased	ERM, cystoid retinal edema, IOP elevation, recurrent CNV	[68]

## 4. Safety and Efficacy of RPE Transplantation

### 4.1. Immune Safety

Clinical trials of RPE transplantation have primarily focused on early-phase evaluations (Phase I and Phase II) to assess safety and efficacy. Among the key concerns in evaluating the feasibility of RPE replacement therapy are immune rejection and surgical complications. The subretinal space, where the transplantation occurs, is considered an immune-privileged site, and RPE cells within this environment possess immunosuppressive properties [69]. Consequently, RPE transplantation demonstrates a relatively high tolerance to immune rejection. Preclinical studies have shown that RPE cells can modulate inflammatory immune responses in vitro and in animal models [70,71]; however, they can also undergo immunogenic transformation, particularly under inflammatory conditions, making them susceptible to immune cell attacks [72]. Notably, clinical studies have reported no significant indications of transplant rejection for ESC-RPE and allogeneic iPSC-RPE [24,47,48,58,64,66,73]. However, stem cell RPE grafts may undergo a slow functional deterioration or rejection without manifest signs of inflammatory reaction. Allogeneic transplants may have relatively high rates of rejection and inflammation [74]. For autologous replacement, no rejection signs were observed in patients receiving autologous iPSC-RPE transplants, which aligns with expectations [29,38].

Evidence supporting the absence of rejection primarily stems from multimodal imaging findings, including OCT and fluorescein angiography, which can detect inflammatory changes such as edema or exudative leakage. Some clinical trials reported heterogeneous reflectance from the RPE layer around the implantation site, which remained stable over long-term follow-up [42,64]. Although the articles did not discuss in depth the causes of these highly reflective substances, this appearance may be a manifestation of macrophage or microglia activation and aggregation in eyes with AMD that are related to the development of the disease [75]. Therefore, it remains challenging to determine the presence of low-grade immune rejection solely through clinical imaging.

A post-mortem histological analysis of a patient who underwent subretinal allogeneic human ESC-derived RPE transplantation two years previously revealed immune cell infiltration, with an increased presence of CD68+ macrophages distributed in the retina and choroid of the transplanted eye. CD8+ cytotoxic T cells were concentrated in the choroid adjacent to the implant and Bruch’s membrane [76]. Given the allogeneic nature of the transplant, total human leukocyte antigen (HLA) matching was not possible; however, no specific antibodies against HLA class I or II molecules from the transplanted cells were detected in the patient’s peripheral blood [42].

It is important to note that, apart from autologous cell-based RPE transplantation, most clinical trials have employed postoperative immunosuppressive therapy. The duration of immunosuppression has varied, with most patients requiring only short-term treatment. In one clinical trial, immunosuppressive therapy was extended to approximately 9 months post-transplantation [73]. Adverse effects associated with immunosuppression are generally mild to moderate and can be effectively managed. Based on the current results, we can be sure that RPE transplantation is relatively safe in terms of immune rejection with the assistance of limited immunosuppressive therapy.

### 4.2. Surgical Complications

Infectious endophthalmitis is the most severe postoperative adverse event associated with RPE transplantation [77]. Only one case of acute infectious endophthalmitis has been reported after RPE transplantation: it occurred four days postoperatively and was successfully treated with antibiotics [22]. The infection was attributed to *Staphylococcus epidermidis*, likely introduced during the surgical procedure rather than originating from the hESC-RPE implant, since tests (including cultures and Gram stains) of the implanted cells showed no signs of bacterial contamination. Other reported surgical complications include retinal or vitreous hemorrhage, retinal detachment, epiretinal membrane formation, cataract development, and transient intraocular pressure (IOP) elevation [20,24,42,58]. The incidence of these complications has decreased with advances in surgical techniques. Notably, the steep learning curve associated with RPE–choroid patch graft procedures has been addressed through experience; in the last 49 transplanted eyes, operating time was nearly halved, and the occurrence of complications was significantly reduced [20].

Compared to the scaffold-based RPE patch implant, hESC-derived RPE cell suspension delivery can be complicated by cell migration to other parts of the eyes, such as preretinal membranes and pigmented deposits in the vitreous [57]. Increased injection pressure during the injection process can lead to cell reflux, which reduces the efficiency of RPE cell delivery. Researchers have developed advanced techniques and surgical instruments aimed at optimizing the injection process to address this issue. These include precise control of injection pressure (10 mmHg) to minimize turbulence and real-time intraoperative OCT for accurate positioning of the injection cannula [58,66]. These innovations help ensure targeted cell delivery and reduce the likelihood of RPE reflux. Long-term follow-up indicates that these migrated cells do not proliferate excessively or impair visual function.

CNV has been observed in approximately 4.5% of cases following RPE–choroid patch transplantation [20]. CNV was typically detected at the patch’s edge and was attributed to intraoperative trauma to the choroid. Similarly, development of CNV postoperatively was reported in one patient with dry AMD following hESC-RPE transplantation; the lesion was localized at the superior margin of the subretinal injection site and may have resulted from either natural disease progression or the mechanical disruption of Bruch’s membrane during the injection procedure [58]. Efforts to reduce surgical complexity remain a key challenge for future RPE transplantation. The development of advanced surgical equipment, improved delivery systems [63], and the use of intraoperative OCT [78] have significantly refined surgical techniques, enabling more precise implantation with minimal trauma.

### 4.3. Improvement and Stability of Vision

VA and overall visual function are the primary measures used to evaluate the effectiveness of RPE transplantation. Clinical trials have employed a variety of visual function tests, including visual field analysis, microperimetry, visual electrophysiology, different types of electroretinograms, contrast sensitivity, color vision assessments, and reading speed evaluations. However, due to inconsistencies in the test methods across studies and the generally poor baseline vision of participants, many visual function tests are unreliable for these eyes. Therefore, in this review, we primarily focus on best-corrected visual acuity (BCVA), which remains the most direct and widely used measure to assess transplant outcomes.

More than half of the studies indicate that RPE transplantation can result in measurable vision improvement [20,24,42,48,65,73], while others suggest that vision is largely maintained post-transplantation. One study reported that the rate of vision loss following RPE transplantation was significantly lower than the natural progression of the disease (a decrease of 7 letters vs. 14 letters over a 3-year follow-up) [42]. In patients with geographic atrophy (GA) who had no prior treatment options, the adjusted mean decrease in BCVA was 13.9 letters in the worse-seeing eye compared to 7.6 letters in the better-seeing eye [79].

Several mechanisms have been proposed to explain the observed stabilization or improvement in vision following RPE transplantation: 1. Paracrine Effects: Transplanted RPE cells secrete growth factors and cytokines that promote photoreceptor survival and maintenance. RPE transplantation can lead to the recovery of some photoreceptor function, particularly if the RPE damage is not too extensive or if the photoreceptor cells themselves are not irreversibly damaged [80]. This effect was evident in a clinical trial in which the neural retina after transplantation was thicker than that of the non-treated eye [73]. Some clinical trials have reported that the neuroretina was thicker in areas overlying the RPE transplant than in areas without a transplant. Ahluwalia et al. demonstrated that the polarized RPE secretome can protect photoreceptors in preclinical models of retinal degeneration [81]. 2. Nutritional and Metabolic Support: Implanted RPE cells facilitate the transport of nutrients and metabolic waste within the subretinal space, thereby rescuing photoreceptors that depend on glucose and energy exchange via the RPE [82]. 3. Surgical Stimulation: The transplantation procedure itself may trigger the release of cytokines and neurotrophic factors, potentially enhancing the residual function of photoreceptors [66]. In three clinical trials involving wet AMD, where CNV was surgically removed before RPE transplantation, improvements in vision were observed [20,60,65]. However, a meta-analysis suggested that CNV removal alone does not contribute significantly to visual improvement in AMD patients [83].

### 4.4. Factors Influencing Vision Outcome

#### 4.4.1. Patient Selection

Most clinical trials involved participants with severely impaired baseline vision; typically, the BCVA was below 20/200. In cases where both eyes were affected by macular degeneration, the eye with poorer vision was selected for treatment. Photoreceptors are often already degenerate in advanced AMD and Stargardt disease [84], making it challenging to achieve substantial visual improvement even if the transplanted RPE cells are functional. Meta-analyses have reported a significantly greater increase in b-wave amplitude and enhanced performance in vision-based behavioral experiments after RPE transplantation in younger animal models of AMD [26]. Additionally, the pattern of vision improvement in young people may be different from that of the elderly [20]. A study by Humayun et al. stratified patients into two cohorts based on baseline VA: those with VA < 20/200 (Cohort 1) and those with VA between 20/80 and 20/400 (Cohort 2). After a 3-year follow-up, Cohort 2 demonstrated a higher proportion of vision improvement (33% vs. 17%) and stabilization, with a lower rate of visual decline (44% vs. 50%) compared to Cohort 1 [42]. These findings suggest that selecting patients with better baseline vision or earlier-stage disease may yield more favorable outcomes in future trials. However, differences in VA testing methodologies (LogMAR vs. Snellen charts) and the learning curve associated with low-vision assessments may also impact the accuracy of results over time [85]. Despite these challenges, the available data suggest that RPE transplantation holds potential for preserving and restoring vision.

#### 4.4.2. Transplantation Site

The site of RPE cell transplantation has varied across studies. Some investigations have employed direct transplantation under the fovea, aiming to rescue remaining photoreceptors and improve central vision [64]. However, in AMD, the fovea is often the most severely damaged region, with extensive photoreceptor loss and significant atrophy of Bruch’s membrane and choriocapillaris [86]. Another consideration is that complete macular atrophy does not mimic the state of the macula in earlier stages of degeneration: it is possible that the greatest therapeutic benefit may come from early treatment when the overlying photoreceptors are still viable [24]. As a result, some researchers advocate for transplanting cells into the transition zone between the atrophic and non-atrophic retina, where residual photoreceptors may still benefit from the support of transplanted RPE cells [57,65]. Different implant sites will have an impact on the final VA assessment, but which one is more likely to save vision still needs more exploration.

#### 4.4.3. The Number of Transplanted Cells

Clinical trials have reported a wide range in the number of transplanted cells, varying from thousands to 1,000,000. A lower number of transplanted cells may be insufficient to fully cover the damaged RPE area and may fail to provide adequate cell signaling to support photoreceptors. Preclinical studies have demonstrated that RPE cell transplantation restores visual function in a dose-dependent manner. Specifically, high doses of up to 200,000 RPE cells derived from hESCs were associated with greater visual function restoration and long-term preservation of rod and cone photoreceptors in the Royal College of Surgeons’ (RCS) rat model [87]. Another study found that increasing transplanted cell concentrations from 5000 to 50,000 resulted in improved functional vision rescue of RCS rats, as assessed by both VA and luminance threshold responses. However, no significantly increased benefit was observed using more cells, suggesting that there is a level of tolerance regarding the number of cells introduced [23].

Despite these promising preclinical findings, clinical trials have not demonstrated a significant difference in visual outcomes based on the number of implanted cells. In one study, patients were divided into four different cell transplant groups, yet only the group receiving 150,000 cells showed variable improvements in VA, with enhancements observed in 3 out of 8 eyes, while no significant vision improvement was reported in the other groups [57]. Schwartz et al. administered three different doses of RPE cells in patients with STGD and AMD but did not report any significant differences in visual outcomes among these groups [24].

There remains a substantial discrepancy between clinical and animal studies regarding the number of transplanted cells and the subsequent outcomes. Nonetheless, recent advances in cell sheet or patch implant transplantation techniques may offer a potential solution to the challenge of optimizing visual improvement in relation to cell dosage.

#### 4.4.4. Long-Term Follow-Up

A common limitation across all current studies is the relatively short observation period, with only a few reporting follow-up durations exceeding five years. Long-term evaluation is crucial, as clinical outcomes may evolve over time. One study reported that the visual fixation point was located at the center of the patch one year after hESC-RPE implantation, but the fixation point had shifted beyond the patch boundary by the third year, coinciding with the appearance of extensive pigmentation. A similar trend was observed in retinal sensitivity measured by microperimetry—while all subjects had improved retinal sensitivity one year post-transplantation, none exhibited sustained improvement compared to baseline after 5 years [48]. On the other hand, it has been reported that some patients who have received autologous RPE–choroidal graft transplantation who have been followed for 6–10 years experienced sudden, unexpected visual improvement more than 1 year after transplantation.

## 5. Challenge and Future Directions of RPE Transplantation

### 5.1. Cell Survival and Integration: Histological Evidence

The survival and functional integration of transplanted RPE cells are critical determinants of the success of cell-based therapies for degenerative retinal diseases. These factors directly influence graft functionality, host-tissue integration, and visual outcomes. Ideally, transplanted RPE cells should survive and maintain their function over the long term. However, defining the survival and functionality of RPE cells in vivo remains challenging, as obtaining direct histological evidence from patients is not feasible.

To date, only one study has assessed RPE survival using post-mortem tissue analysis. Immunofluorescence staining of post-mortem tissue from an eye that received an RPE transplant revealed the presence of RPE cell polarity and functional markers, including RPE65, BEST1, and Na^+^/K^+^ ATPase. Additionally, phagosomes containing photoreceptor outer segments were observed in donor RPE cells, along with staining of native choroidal vessels beneath Bruch’s membrane, indicating integration with both the photoreceptors and choriocapillaris. However, the RPE implant, which had been placed on a parylene membrane, struggled to maintain a coherent monolayer structure, and elevated levels of CD68 and CD8 markers suggested persistent immune responses even two years post-transplantation [76]. This finding underscores the potential need for long-term immunosuppression to ensure prolonged graft survival.

Despite the scarcity of human histological data, numerous preclinical studies have demonstrated that donor RPE cells can survive and express relevant biomarkers in the subretinal space of various animal models. These findings provide valuable insights into the potential longevity and integration of transplanted RPE cells [28,88,89].

### 5.2. Clinical Assessment of RPE Cell Survival

OCT and fundus autofluorescence have emerged as valuable non-invasive imaging modalities that provide real-time insights into the structural integrity and persistence of transplanted RPE cells. Regardless of the cell source—whether autologous, hESC-RPE, or iPSC-RPE—researchers commonly use the formation and persistence of pigmentation as an indicator of RPE cell survival [49]. When fundus images are combined with OCT segmentation, the persistence and expansion of pigmentation are often interpreted as evidence of transplanted RPE survival, exhibiting similar characteristics to native RPE [29,38]. A recent study by Lyndon et al. reported that, during a 5-year follow-up, the thickness of the transplanted hESC-RPE cell layer remained at approximately 15 μm on OCT imaging [48], indirectly suggesting that the transplanted cells did not degenerate. But it could not prove the transplanted cells were retained in their morphology.

However, pigmentary changes such as migration and hyperpigmentation may not exclusively indicate the presence of RPE cells; they could also result from macrophage phagocytosis or pigment efflux following RPE degeneration [90]. Retinal electrophysiological tests, such as electro-oculography (EOG) and electroretinography (ERG), are theoretically useful tools to assess RPE function and survival indirectly. However, clinical trials have largely reported that EOG and ERG results show no significant changes post-transplantation, and findings do not always correlate with improvements in vision. This discrepancy may be attributed to several factors, including the poor baseline vision of patients and their limited ability to fully cooperate during testing.

To enhance the reliability of functional assessments, researchers are exploring combinatorial approaches, integrating multimodal imaging with functional tests to provide a more comprehensive evaluation of transplanted RPE survival and integration [91]. The accumulation of clinical trial data and continued advances in imaging technologies are expected to facilitate better monitoring and assessment of RPE graft viability in the future.

### 5.3. Factors That Influence Cell Survival

Several factors influence the survival and integration of transplanted RPE cells, with cell source, delivery methods, and the retinal microenvironment playing crucial roles.

While each RPE source offers distinct advantages and limitations, clinical studies have yet to identify the optimal cell source for achieving long-term survival in human eyes. A systematic review of 124 preclinical studies in animal models reported that OpRegen^®^ RPE cells, derived from ESCs, successfully survived in the subretinal space and improved vision [26]. Other studies have indicated that hESC- and iPSC-derived RPE cells exhibit similar behavior in terms of survival, migration, integration, and functional support of photoreceptors in the RCS rat model of retinal degeneration [92]. However, iPSC- RPE cells have been shown to exhibit higher immunogenic potential than ESC-RPE cells, which could affect their long-term survival. Additionally, the genomic instability associated with iPSCs poses safety concerns for their clinical application [93]. For instance, researchers detected three DNA copy number deletions during cell preparation for a clinical trial involving autologous iPSC-RPE transplantation that might have affected gene expression [38].

Studies suggest that polarized monolayers of hESC-RPE survive better than cell suspensions [94]. Polarized monolayers exhibit greater resistance to oxidative stress-induced cell death, whereas nonpolarized cultures experience higher rates of apoptosis with increased expression of pro-apoptotic factors under similar conditions [95]. While RPE cell suspensions require 1–2 weeks to fully differentiate and integrate within the retinal environment, scaffold-based RPE implants can immediately provide trophic support to photoreceptors and choroidal endothelial cells, establishing an immune-suppressive environment in the subretinal space [96].

However, a meta-analyses of RPE transplant in animal models found no significant difference in the effect of an RPE sheet versus an RPE suspension transplantation for both the b-wave amplitude and the vision-based behavior assays [26]. Subretinal fibrosis has been reported in some cases of scaffold-based RPE delivery, which could potentially affect long-term visual outcomes [60,97]. As mentioned above, RPE suspension cell injection is simpler than RPE monolayer transplantation and causes less surgical damage. In the future, as surgical techniques advance, the development of less invasive and more precise delivery methods may enhance the survival of polarized RPE patches.

The subretinal microenvironment plays a critical role in determining transplant success. Sandra et al. reported that hESC-RPE cells successfully integrated as subretinal monolayers in non-pretreated naive eyes with preserved neuroretina but failed to integrate in areas with advanced neuroretinal degeneration and RPE loss [98]. These findings underscore the importance of a preserved outer neuroretina/RPE complex for the successful integration of donor-derived hESC-RPE cells. Studies have also shown that the ESC microenvironment promotes human RPE cell proliferation by activating the PI3K signaling pathway, which may improve survival rates [99]. Animal models further suggest that RPE transplantation yields better outcomes in younger subjects, highlighting the potential benefits of earlier intervention in patients with retinal degenerative diseases [26]. Optimizing the local microenvironment during transplantation or selecting patients in earlier disease stages represents a promising strategy to improve the efficacy of RPE transplantation in the future.

### 5.4. Enhancing RPE Survival and Future Directions

Researchers are exploring molecular and genetic strategies to enhance the survival and integration of transplanted RPE cells. Croze et al. demonstrated that Rho-associated kinase (ROCK) inhibition significantly enhances attachment, proliferation, and wound closure in human ESC-RPE cultures [100]. In vivo studies using the ROCK inhibitor Y-27632 increased transplant viability, suppressed apoptosis, and enhanced cell adhesion of iPSC-RPE transplants in the subretinal space of monkeys without significant retinal toxicity [101]. It is important to note that ROCK inhibition not only preserves the stem cell phenotype but also affects metabolic pathways such as glycolysis, glutaminolysis, and the citric acid cycle, which could have long-term implications [102].

The development of structurally and functionally biomimetic scaffold membranes has become a major research focus to improve biocompatibility and functional integration. hAM has good biocompatibility, mechanical support, and nutrient permeability, and is already used in various retinal surgeries [103]. Notably, a monolayer of iPSC-derived RPE (iPSC-RPE) cultured on a hAM-based scaffold was approved to initiate a Phase I/II clinical trial in 2019 [67]. In parallel, other biodegradable PLGA scaffolds have demonstrated favorable safety profiles in preclinical studies, showing no inflammatory response and minimal impact from degradation byproducts on retinal tissue [50]. To further enhance scaffold performance, biomaterials incorporating components of Bruch’s membrane, such as collagen, are under investigation. These materials can be engineered using advanced biofabrication techniques to optimize both physical properties and biological interactions [55]. Emerging approaches now combine RPE with photoreceptor precursors on engineered scaffolds. For instance, micromolded honeycomb-patterned PGS scaffolds enable dual-layer RPE+ photoreceptor constructs, which maintained spatial structure and stability following transplantation in animal models [104]. Nonetheless, larger-scale and long-term studies are still needed to fully validate the safety, durability, and therapeutic efficacy of these next-generation biomaterials.

Genetic modification of RPE cells is another promising avenue for improving transplant outcomes. A novel long noncoding RNA (lncRNA), TREX, was identified as a potential biomarker for transplant success in the RCS rat model. Overexpression of TREX significantly improved cell integration and enhanced vision recovery, while its knockdown reduced transplant efficacy [105]. Additionally, the transplantation of RPE cells genetically modified to express OTX2, a critical transcription factor for RPE development, has shown promise in treating retinal diseases such as AMD [106].

Continued research efforts focusing on improving the cellular microenvironment, optimizing delivery techniques, and leveraging genetic modifications hold the potential to significantly enhance the survival and functional integration of transplanted RPE cells. Advances in biomaterials, imaging techniques, and minimally invasive surgical procedures are expected to further refine RPE transplantation as a viable therapeutic option for retinal degenerative diseases.

## 6. Conclusions

RPE transplantation represents a promising therapeutic strategy for retinal degenerative diseases such as AMD and Stargardt disease, offering the potential to restore critical retinal functions and improve visual outcomes. Over recent decades, advancements in cell engineering, surgical techniques, and biomaterials have propelled this field from experimental models to clinical applications. Clinical trials have demonstrated the feasibility and relative safety of RPE transplantation, with no significant immune rejection when appropriate immunosuppressive regimens are employed.

Variability in surgical methods, differences in cell sources, and limitations in follow-up duration have led to inconsistent efficacy outcomes so far. Meanwhile, several challenges remain, including optimizing cell survival, enhancing host integration, and overcoming immune responses. Additionally, the transplantation site and the patient’s disease stage significantly influence treatment outcomes, emphasizing the need for personalized approaches.

Future research is warranted to refine transplantation techniques, including minimally invasive delivery systems and scaffold-based support structures, exploring alternative cell sources and optimizing differentiation protocols to produce more functional and disease-resistant RPE cells, as well as preconditioning strategies to enhance the subretinal microenvironment. Advances in imaging modalities and functional assessments will further aid in monitoring graft survival and integration. Furthermore, the integration of molecular strategies and genetic modifications holds promise in enhancing the longevity and function of transplanted RPE cells.

In conclusion, while RPE transplantation has shown significant potential, addressing the current challenges through multidisciplinary approaches and longer-term clinical evaluation is needed for the treatment to fully realize its therapeutic potential. With continued advances in stem cell technology, surgical innovations, and patient selection criteria, RPE transplantation is poised to become an effective and accessible treatment for patients suffering from vision loss due to retinal degeneration.

## Figures and Tables

**Table 1:** Different cell sources and their features of RPE transplantation.

RPE Cell Source		Strengths	Weaknesses
Native RPE	Fetal RPE	A certain level of plasticity and regeneration; more closely mimics developmental stage-specific functions	Limited availability; ethical concerns over fetal tissue use; immune rejection risk
	Adult RPE	Native morphology and gene expression; lower tumorigenicity risk; ethical acceptability	Scarce availability; limited proliferative capacity; variant quality of cells; high risk of immune rejection in non-autologous transplants
Stem cell-derived RPE	hESC-RPE	Unlimited supply; high regenerative and proliferative potential; uniformity and scalability for standardizing therapeutic applications	Ethical concerns regarding use of embryonic cells; risk of tumorigenicity; mild to moderate immune rejection dependent on immunosuppressants manufacturing complexity
	iPSC-RPE	Unlimited supply; high regenerative and proliferative potential; ethical acceptability; Patient-specific applications; genetic correction potential	complex manufacturing process; potential tumorigenicity; potential genomic instability; high cost of patient-specific derived cells; immune rejection risk in allogeneic transplantation

**Table 2:** Different delivery methods and their comparison.

RPE Transplant Delivery	Cell Amount and Implant Area	Advantages	Disadvantages
Cell suspension	Cells from native 0.5–10 × 10^5^ cells in 0.1–0.15 mL	Simple, minimally invasive, adaptable	Cell survival issue, migration risk, low integration efficiency
RPE–choroid patch	Depends on lesion area, approximately three optic disc diameters	Natural structure, stable placement, native-like function	Complex surgery, donor limitations, retina damage at periphery
RPE cell sheet	1 × 10^5^ cells in 1.3–2 × 2–3 mm	Mimics native RPE, functional integration, reduce foreign material	Fragility, difficult handling, cell detachment risk
Scaffold-based implant	1–10 × 10^5^ cells in 3–3.5 × 6–6.25 mm	Stability, customizability, controlled placement	Foreign material immune reaction, complex production, potential barriers

Details on cell number and implant area in clinical trials can be found in Table 3.

## Data Availability

No new data were created or analyzed in this study.

## Abbreviations

The following abbreviations are used in this manuscript:

RPE	Retinal pigment epithelium
AMD	Age-related macular degeneration
STGD	Stargardt disease
hESC-RPE	Human embryonic stem cell-derived RPE
iPSC-RPE	Induced pluripotent stem cell-derived RPE
RCS	Royal College of Surgeons
OCT	Optical coherence tomography
PLGA	Poly lactic-co-glycolic acid
hAM	Human amniotic membrane
HLA	Human leukocyte antigen
IOP	Intraocular pressure
CNV	Choroidal neovascularization
BCVA	Best-corrected visual acuity
GA	Geographic atrophy
VA	Visual acuity
EOG	Electro-oculography
ERG	Electroretinography
ROCK	Rho-associated kinase
